# Induction of Neuroinflammation and Neurotoxicity by Synthetic Hemozoin

**DOI:** 10.1007/s10571-019-00713-4

**Published:** 2019-07-22

**Authors:** Ravikanth Velagapudi, Ayokulehin M. Kosoko, Olumayokun A. Olajide

**Affiliations:** 1 Department of Pharmacy, School of Applied Sciences, University of Huddersfield, Queensgate, Huddersfield, HD1 3DH UK; 2 Present Address: Department of Anesthesiology, Center for Translational Pain Medicine, Duke University Medical Center, Durham, NC 27710 USA

**Keywords:** Hemozoin, Neuroinflammation, Neurodegeneration, NF-κB, BV-2 microglia, Neural progenitor (ReNcell VM) cells

## Abstract

Hemozoin produced by *Plasmodium falciparum* during malaria infection has been linked to the neurological dysfunction in cerebral malaria. In this study, we determined whether a synthetic form of hemozoin (sHZ) produces neuroinflammation and neurotoxicity in cellular models. Incubation of BV-2 microglia with sHZ (200 and 400 µg/ml) induced significant elevation in the levels of TNFα, IL-6, IL-1β, NO/iNOS, phospho-p65, accompanied by an increase in DNA binding of NF-κB. Treatment of BV-2 microglia with sHZ increased protein levels of NLRP3 with accompanying increase in caspase-1 activity. In the presence of NF-κB inhibitor BAY11-7082 (10 µM), there was attenuation of sHZ-induced release of pro-inflammatory cytokines, NO/iNOS. In addition, increase in caspase-1/NLRP3 inflammasome activation was blocked by BAY11-7082. Pre-treatment with BAY11-7082 also reduced both phosphorylation and DNA binding of the p65 sub-unit. The NLRP3 inhibitor CRID3 (100 µM) did not prevent sHZ-induced release of TNFα and IL-6. However, production of IL-1β, NO/iNOS as well as caspase-1/NLRP3 activity was significantly reduced in the presence of CRID3. Incubation of differentiated neural progenitor (ReNcell VM) cells with sHZ resulted in a reduction in cell viability, accompanied by significant generation of cellular ROS and increased activity of caspase-6, while sHZ-induced neurotoxicity was prevented by *N*-acetylcysteine and Z-VEID-FMK. Taken together, this study shows that the synthetic form of hemozoin induces neuroinflammation through the activation of NF-κB and NLRP3 inflammasome. It is also proposed that sHZ induces ROS- and caspase-6-mediated neurotoxicity. These results have thrown more light on the actions of malarial hemozoin in the neurobiology of cerebral malaria.

**Electronic supplementary material:**

The online version of this article (10.1007/s10571-019-00713-4) contains supplementary material, which is available to authorized users.

## Introduction

Microglia-mediated neuroinflammation is now associated with the pathogenesis of a wide range of brain disorders resulting in the release of neurotoxic chemicals which produce adverse effects on adjacent neurons. In this context, several animal and human studies have proposed that serum concentrations of pro-inflammatory cytokines and chemokines correlate with the severity of cerebral malaria, a parasitic infection caused by *Plasmodium falciparum* thus suggesting the role of neuroinflammation in the disease (Jaramillo et al. 2004; Ioannidis et al. 2014). Expectedly, a high incidence of neurocognitive impairment was reported in cerebral malaria (Idro et al. 2005; Murray et al. 2012). This cognitive deficit has been linked to parasite sequestration in the CNS and activation of inflammatory responses mediated by neurotoxic factors such as nitric oxide (NO), tumor necrosis factor alpha (TNFα), gamma interferon (IFNγ), interleukin-6 (IL-6) and interleukin-1β (IL-1β) which damage adjacent neurons (Brown et al. 1999; McCall and Sauerwein 2010; Sahu et al. 2013). These observations have been supported by animal data and experiments conducted on post-mortem human brain which showed the involvement of TNFα and IFNγ in the development of cerebral malaria (Grau et al. 2003; Wiese et al. 2006). There have been suggestions that brain-resident macrophages, the microglia, are the source of neurotoxic-soluble factors such as nitric oxide and the pro-inflammatory cytokines in cerebral malaria (Szklarczyk et al. 2007), and their activation contributes to the neurological sequelae in the disease. Interestingly, a study by Guha et al. (2014) showed that a single episode of mild malaria produced alterations in the levels of pro-inflammatory cytokines in the brain hippocampus, accompanied by hippocampal microglial activation. There have also been reports showing that autopsy materials of patients who died of cerebral malaria, as well as brain of mice with cerebral malaria contain activated microglia (Medana et al. 2002; Wiese et al. 2006).

Considering the role of neuroinflammation in CNS pathologies, an understanding of the mechanisms underlying its activation in cerebral malaria will help in developing new adjunctive treatments for the disease.

It is believed that a brown crystalline by-product formed during the catabolism of hemoglobin by the malarial parasite, known as hemozoin (HZ), is released into the systemic circulation and accumulates into insoluble crystals inside the host red blood cells. There have been suggestions that HZ could play a pivotal role in cerebral malaria pathophysiology related to cytokine overproduction in the brain (Coronado et al. 2014). In the periphery, malaria HZ has been shown to induce innate immunity and inflammation through the release of TNFα, IL-1β, and chemokines such as macrophage inflammatory protein-1α (MIP-1a/CCL3), macrophage inflammatory protein-1β (MIP-1β/CCL4), macrophage inflammatory protein-2 (MIP-2/CXCL2), monocyte chemoattractant protein (MCP)-1/CCL2, and chemokine receptors such as CCR1, CCR2, CCR5, CXCR2, and CXCR4, as well as NO and reactive oxygen species (Shio et al. 2010). HZ-induced inflammation has been linked to a number of mechanisms including NLRP3 inflammasome-mediated IL-1β and IL-18 production (Griffith et al. 2009; Dostert et al. 2009), as well as NOD2-mediated NO production (Corbett et al. 2015).

In light of the role played by neuroinflammation in cerebral malaria and reports linking HZ to innate immunity and inflammation in the periphery, we have investigated synthetic hemozoin-induced neuroinflammation in BV-2 microglia. We also evaluated the potential neurotoxicity produced by the pigment in a 3D human neural cell model.

## Materials and Methods

Synthetic hemozoin (sHz) was purchased from InvivoGen (Toulouse, France). This was prepared in sterile water and sonicated prior to use.

### Cell Culture

BV-2 mouse microglia cell line ICLC ATL03001 (Interlab Cell Line Collection, Banca Bilogica e Cell Factory, Italy) was maintained in RPMI1640 medium (Gibco) with 10% fetal bovine serum (FBS) (Sigma), 2 mM l-glutamine (Sigma), 100 U/ml penicillin, and 100 mg/ml streptomycin (Sigma) in a 5% CO_2_ incubator.

ReNcell VM is an immortalized human neural progenitor cell line with the ability to readily differentiate into mature neurons and glial cells (oligodendrocytes and astrocytes). These cells provide an excellent model for investigating substances that have the potential to cause damage to mature functional human neurons. ReNcell VM cells were acquired commercially from Millipore (Hertfordshire, UK). Cells were maintained on laminin-coated 75 cm^3^ culture flask (Sarstedt, UK) in ReNcell NSC maintenance medium (Millipore, UK) containing freshly prepared epidermal growth factor (20 ng/ml; Gibco) and fibroblast growth factor-2 (20 ng/ml; Gibco). Differentiation of these cells was initiated by removing the medium from each well and replaced with fresh ReNcell NSC Maintenance Medium that did not contain FGF-2 and EGF (Velagapudi et al. 2018).

### Release of Nitrite and Pro-Inflammatory Cytokines from BV-2 Microglia

BV-2 cells were cultured for 48 h, followed by stimulation with sHZ (200 and 400 µg/ml) for 24 h. Concentrations of nitrite in culture media were measured using commercially available Griess assay kit (Promega) according to the manufacturer’s instructions. Absorbance was measured at 540 nm in a microplate reader (Infinite F50, Tecan). Concentrations of TNFα, IL-6, and IL-1β were measured using mouse ELISA kits (BioLegend, UK), followed by measurements in a plate reader at a wavelength of 450 nm. Levels of pro-IL1β were detected using mouse pro-IL1β ELISA kit (Invitrogen). These experiments were also carried out in sHZ-stimulated BV-2 microglia pre-treated with the NF-κB inhibitor Bay 11-7082 (10 µM), and the NLRP3 inflammasome inhibitor, CRID3 (100 µM).

### BV-2 Microglia-ReNcell VM Co-culture

BV-2 microglia-neuron co-culture experiments were done to determine whether sHZ-induced neuroinflammation would induce damage to adjacent neural structures. Co‐culture experiment was carried out using transwell system (Corning), employing a membrane with 0.4 µm pore size. Differentiated ReNcell VM cells (1 × 10^5^) were cultured in the lower chamber, while 5 × 10^4^ BV-2 microglia were cultured in the upper chamber. Following the establishment of the co-culture, BV-2 microglia in the upper chamber were incubated with sHZ (200 and 400 µg/ml) for 24 h. After the incubation period, the viability of differentiated ReNcell VM cells was determined using MTT assay. Supernatants were collected from the co-culture and levels of nitrite, TNFα, and IL-1β measured. To control for the impact of sHZ diffusing across the transwell membranes and affecting ReNcell VM cells directly, wells containing ReNcell VM cells were co-cultured with inserts (with or without BV-2 microglia). Thereafter, viability of ReNcell VM cells co-cultured with BV-2 microglia was compared with those co-cultured without BV-2 microglia.

### Caspase-Glo^®^ 1 Inflammasome Assay

In neuroinflammation, activation of the NLRP3 inflammasome in turn results in the activation of caspase-1, which then promotes the processing and release of IL-1β. The Promega Caspase-Glo^®^ 1 inflammasome assay is a reliable method for measuring the activity of caspase-1. The assay employs a luminogenic caspase-1 substrate, Z-WEHD-aminoluciferin, which has been optimized for caspase-1 and luciferase activities. Addition of this reagent produces cell lysis, substrate cleavage by caspase-1 and generation of light by luciferase. BV-2 microglia were cultured in a 96-well plate at 5.0 × 10^4^ cells/well. Thereafter, cells were incubated with sHZ (200 and 400 µg/ml) for 24 h. At the end of the incubation period, the plate was left to equilibrate at room temperature for 5 min. The assay was carried out according to the manufacturer’s instructions. Luminescence was read with FLUOstar OPTIMA reader (BMG LABTECH).

### NF-κB DNA-Binding Assay

Experiments to evaluate DNA binding of NF-κB, following sHZ treatment were carried out as described earlier (Velagapudi et al. 2017; El-Bakoush and Olajide 2018). Nuclear extracts from BV-2 microglia treated with sHZ (200 and 400 µg/ml) were added into a 96-well plate on which NF-κB consensus site (5′ GGGACTTTCC-3′) has been immobilized (Active Motif, Belgium). Thereafter, the plate was incubated with NF-κB antibody (1:1000) for 1 h, followed by HRP-conjugated antibody (1:1000) for a further 1 h. Absorbance was read on a Tecan F50 microplate reader at 450 nm.

### Western Blotting

Cultured BV-2 microglia were treated with either 200 or 400 µg/ml sHZ for 24 h. Thereafter, protein was extracted from lyzed cells. Electrophoresis was performed by SDS-PAGE to separate the proteins. Proteins were then transferred onto polyvinylidene difluoride (PVDF) membrane (Millipore). Transferred proteins were exposed to rabbit anti-iNOS (1:1000; Cell Signaling), rabbit anti-NLRP3 (1:1000; Abcam), rabbit anti-phospho-p65 (1: 1000; Santa Cruz), and rabbit anti-actin (1:1000; Sigma). Membranes were incubated with the primary antibody overnight at 4 °C. Proteins were detected. Blots were detected with Alexa Fluor^®^ 680 goat anti-rabbit IgG (Life technologies, UK) using Licor Odyssey imager.

### Differentiated ReNcell VM Viability and Generation of Cellular Reactive Oxygen Species

Differentiated ReNcell VM cells were treated with sHZ (200 and 400 µg/ml) and incubated for 24 h. Thereafter, cell viability was assessed using the MTT cell viability assay as earlier described (Velagapudi et al. 2017).

Generation of intracellular reactive oxygen species (ROS) levels in differentiated ReNcell VM neural cells was performed using the fluorescent 2′, 7′-dichlorofluorescin diacetate DCFDA-cellular reactive oxygen species detection assay kit (Abcam). Cells were incubated with 10 µM DCFDA for 30 min at 37 °C. After removal of excess DCFDA, the cells were washed and stimulated with sHZ (200 and 400 µg/ml) for a further 4 h at 37 °C. Intracellular production of ROS was measured by the fluorescence detection of dichlorofluorescein (DCF) as the oxidized product of DCFH on a microplate reader (BMG Labtech) with an excitation wavelength of 485 nm and emission wavelength of 535 nm.

### Caspase-6 Activity Assay

Following differentiation, ReNcell VM cells were treated with sHZ (200 and 400 µg/ml) and incubated for 24 h. After the incubation period, cytosolic extracts were prepared and  2 × reaction buffer (containing 10 mM DTT) was added to the samples. This was followed by the addition of 200 μM of VEID-p-NA substrate (Abcam). Absorbance was read in a Tecan microplate reader (405 nm) following a 2-h incubation at 37 °C. Fold increase in VEID-dependent caspase activity was evaluated.

### Effects of *N*-Acetylcysteine and Z-VEID-FMK on sHZ-Induced Neurotoxicity

Differentiated ReNcell VM cells were pre-treated with the ROS inhibitor (*N*-acetylcysteine; 1 mM) and caspase-6 inhibitor (2 µM) 30 min prior to exposure to sHZ (400 µg/ml) for a further 24 h. Thereafter, cell viability was evaluated using the MTT assay.

### Statistical Analysis

Values of all experiments were represented as a mean ± SEM of at least three experiments. Values were compared using one-way ANOVA followed by a post hoc Tukey’s multiple comparison test, which compared mean values from each treatment with the mean values of every other treatment.

## Results

### sHZ Induces the Production of Pro-Inflammatory Cytokines from BV-2 Microglia

**Fig. 1:**
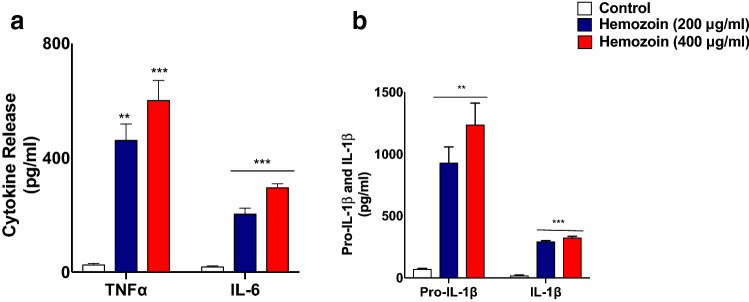
sHZ increased levels of cytokines, pro-IL-1β and IL-1β in BV2 microglia. BV2 cells were treated with 200 and 400 µg/ml of sHZ for 24 h. Culture supernatants were collected and analyzed for **a** TNF-α, IL-6, and **b** pro-IL-1β and IL-1β using ELISA. sHZ increased the concentrations of the cytokines, pro-IL-1β, and IL-1β. All values are expressed as mean ± SEM for three independent experiments. Data were analyzed using one-way ANOVA followed by a post hoc Tukey’s multiple comparison test. **p < 0.01, ***p < 0.001 compared with untreated control

Pro-inflammatory cytokines such as TNFα, IL-6, and IL-1β are some of the principal mediators released by activated microglia. Consequently, we were firstly interested in determining whether these cytokines would be released following the exposure of BV-2 microglia to sHZ. Results showed at least 400-fold elevation in the concentrations of both TNFα and IL-6 following incubation of BV-2 microglia with sHZ (200 and 400 µg/ml) for 24 h (Fig. 1a). The release of IL-1β is known to involve the conversion of pro-IL-1β to the mature form. Consequently, separate experiments were carried out to determine the effects of sHZ on these cytokines. Analyses of culture supernatants obtained from BV-2 microglia incubated with sHZ (200 and 400 µg/ml) for 24 h revealed significant (*p* < 0.01) elevation of both pro-IL-1β and IL-1β (Fig. 1b).

### iNOS Mediates Production of Nitrite by sHZ in BV-2 Microglia

**Fig. 2:**
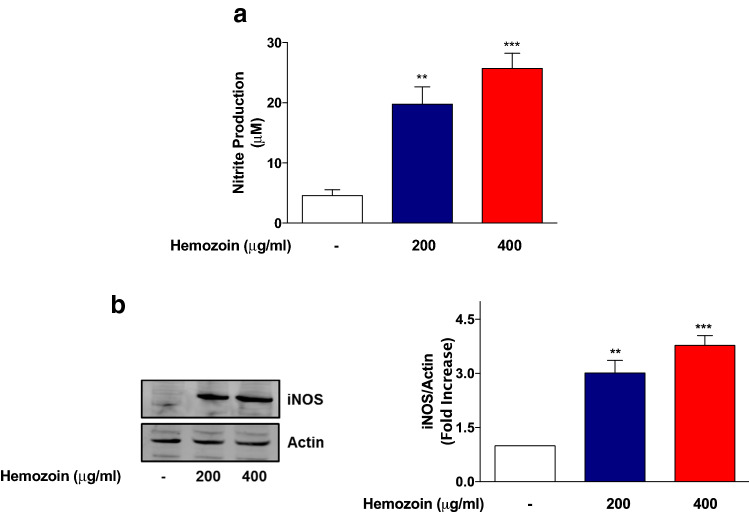
Nitrite production and iNOS protein expression were increased in sHZ-treated microglia. BV2 cells were stimulated with sHZ (200 and 400 µg/ml) for 24 h. Subsequently, culture supernatants and cytoplasmic lysates were collected and analyzed for **a** nitrite production and **b** iNOS protein expression using ELISA and western blot. Actin was used as a loading control. All values are expressed as mean ± SEM for three independent experiments. Data were analyzed using one-way ANOVA with post hoc Tukey’s multiple comparison test. **p < 0.01, ***p < 0.001 compared with untreated control

As shown in Fig. 2a, the concentration of nitrite produced in BV-2 microglia increased significantly (*p* < 0.01) from ~ 4.6 µM in control supernatants to ~ 20 µM following incubation with sHZ (200 µg/ml) for 24 h. On increasing the concentration of sHZ to 400 µg/ml, the level of nitrite in culture supernatants was further increased to ~ 25 µM (Fig. 2a). Similarly, levels of iNOS protein were undetectable in control untreated. However, following the exposure to both concentrations of sHZ, there was marked and significant (*p* < 0.05) increase in the levels of iNOS protein (Fig. 2b).

### sHZ Induced Neuroinflammation-Mediated Damage to Differentiated ReNcell VM Cells

**Fig. 3:**
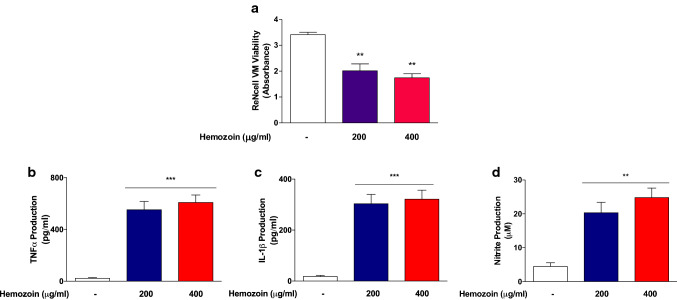
Hemozoin produced neuroinflammation-mediated neural damage in differentiated ReNcell VM human neural cells. **a** Results of MTT assay showing that the viability of neural cells was significantly reduced following 24-hour incubation with sHZ (200 and 400 µg/ml). Loss of neural cells was accompanied by significant increases in the levels of TNFα (**b**), IL-1β (**c**), and nitrite (**d**). All values are expressed as mean ± SEM for three independent experiments. Data were analyzed using one-way ANOVA with post hoc Tukey’s multiple comparison test. **p < 0.01, ***p < 0.001 compared with untreated control

We further determined whether the activation of BV-2 microglia by sHZ could result in neuroinflammation-mediated damage to differentiated ReNcell VM human neural cells using a transwell co-culture system. MTT assay results displayed in Fig. 3a shows that treating BV-2 microglia on the upper layer of the culture with sHZ (200 and 400 µg/ml) resulted in significant (*p* < 0.01) reduction in the viability of ReNcell VM human neural cells in the lower layer. In order to confirm the presence of secreted neurotoxic factors in the co-culture, supernatants were analyzed and results showed significant (*p* < 0.01) elevation in the levels of nitrite (Fig. 3a), TNFα (Fig. 3b), and IL-1β (Fig. 3d) in the co-culture environment, in comparison with control. Initial experiments to control possible diffusion of sHZ across the insert membrane to induce direct neurotoxicity on ReNcell VM cells show that in the absence of BV-2 microglia in the upper layer of the co-culture, sHZ produced minimal effects on the viability of ReNcell VM cells. However, with BV-2 microglia there was a significant (*p* < 0.01) reduction in viability of ReNcell VM cells, in comparison with untreated control, confirming BV-2 microglia-mediated neurotoxicity by the sHZ (Supplementary Data 1).

### sHZ Increases NLRP3 Protein and Caspase-1 Activity in BV-2 Microglia

**Fig. 4:**
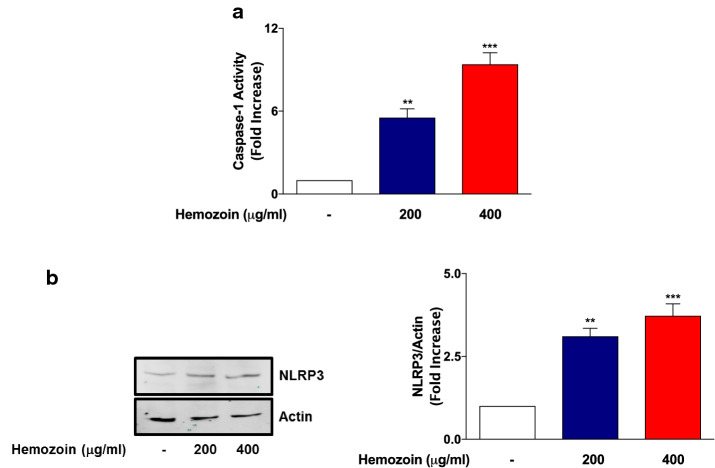
sHZ increases caspase-1 activity and NLRP3 protein expression in BV-2 microglia. BV2 cells were exposed to sHZ (200 and 400 µg/ml) for 24 h. Later, **a** caspase-1 activity was measured using inflammasome Glo assay and **b** cytoplasmic lysates were used to detect NLRP3 expression using western blot. Actin was used as a loading control. All values are expressed as mean ± SEM for three independent experiments. Data were analyzed using one-way ANOVA for multiple comparisons with post hoc Tukey’s multiple comparison test. **p < 0.01, ***p < 0.001 compared with untreated control

Results showing that sHZ could induce the release of pro-IL-1β/IL-1β in BV-2 microglia led us to investigate any role played by the activation of NLRP3 inflammasome and whether these effects are accompanied by an increase in caspase-1 activity. Using the inflammasome Glo assay which measures the activity of caspase-1, we show in Fig. 4a that exposure of BV-2 microglia to sHZ (200 and 400 µg/ml) resulted in significant increases in caspase-1 activity. This observation suggests that sHZ activates caspase-1 in BV-2 microglia, resulting in processing of pro-IL-1β to the mature IL-1β. This is further confirmed by results of immunoblotting experiments given in Fig. 4b, which show that incubation of BV-2 cells with sHZ produced a concentration-dependent increase in NLRP3 protein.

### Treatment of BV-2 Microglia with sHZ Resulted in Activation and DNA Binding of p65 Sub-unit

**Fig. 5:**
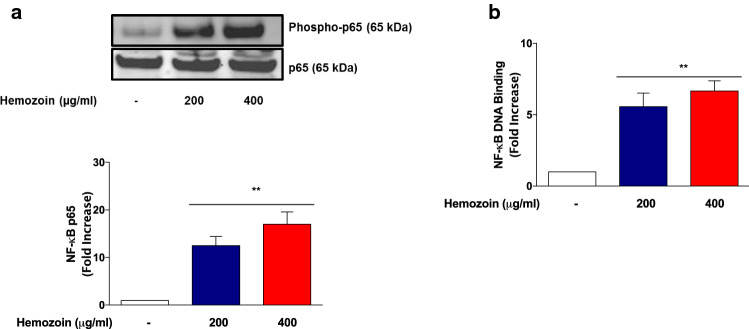
Hemozoin increased neuroinflammation by targeting DNA binding and phosphorylation of NF-κB in the microglia. BV2 cells were stimulated with 200 and 400 µg/ml of sHZ for 60 min. Subsequently, nuclear, and cytoplasmic lysates were collected and subjected to ELISA-based EMSA and western blot. sHZ increased **a** NF-κB DNA binding and **b** phosphorylation of p65 in BV2 microglia. Total p65 was used as a loading control in western blot. All values are expressed as mean ± SEM for three independent experiments. Data were analyzed using one-way ANOVA with post hoc Tukey’s multiple comparison test. **p < 0.01 compared with untreated control

NF-κB is responsible for the transcription of genes encoding pro-inflammatory proteins in the microglia in neuroinflammation. Based on the observation that sHZ induced the production of TNFα, IL-6, IL-1β, and iNOS in BV-2 microglia, we were interested in determining whether exposure to the pigment could activate NF-κB in BV-2 microglia. Results of immunoblotting in Fig. 5a show that in untreated cells, there were minimal levels of phosphorylated NF-κB p65 protein. However, in the presence of sHZ (200 and 400 µg/ml), there was a marked and significant (*p* < 0.01) increase in phosphorylation of NF-κB p65, suggesting an activation of the transcription factor by the pigment in BV-2 microglia. Results of DNA-binding experiments in Fig. 5b show that incubation of BV-2 microglia with sHZ resulted in significant (*p* < 0.01) increase in DNA binding of NF-κB, in comparison with control, suggesting that the effect of the pigment promotes subsequent transcriptional mechanisms resulting in the release of pro-inflammatory proteins.

### The NF-κB Inhibitor BAY11-7082 Prevented Neuroinflammation in sHZ-Stimulated BV-2 Microglia

**Fig. 6:**
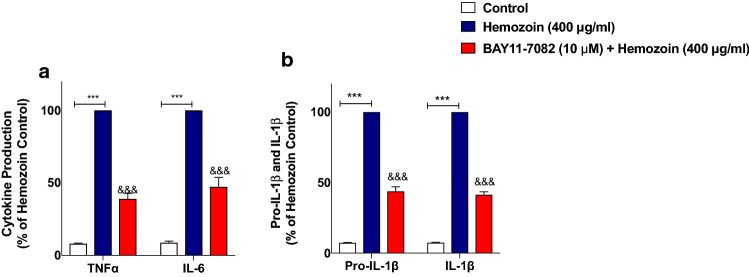
The NF-κB inhibitor BAY11-7082 prevented the release of TNFα, IL-6, and pro-IL-1β/IL-1β in sHZ-stimulated BV-2 microglia. BV2 cells were pre-treated with BAY11-7082 (10 µM) prior to exposing the cells to 400 µg/ml of sHZ. Culture supernatants were collected and analyzed for **a** TNF-α, IL-6, and **b** pro-IL-1β and IL-1β using ELISA. All values are expressed as mean ± SEM for three independent experiments. Data were analyzed using one-way ANOVA followed by a post hoc Tukey’s multiple comparison test. ***p < 0.001 compared with untreated control. ^&&&^*p* < 0.001, Hemozoin alone versus BAY11-7082 + Hemozoin

**Fig. 7:**
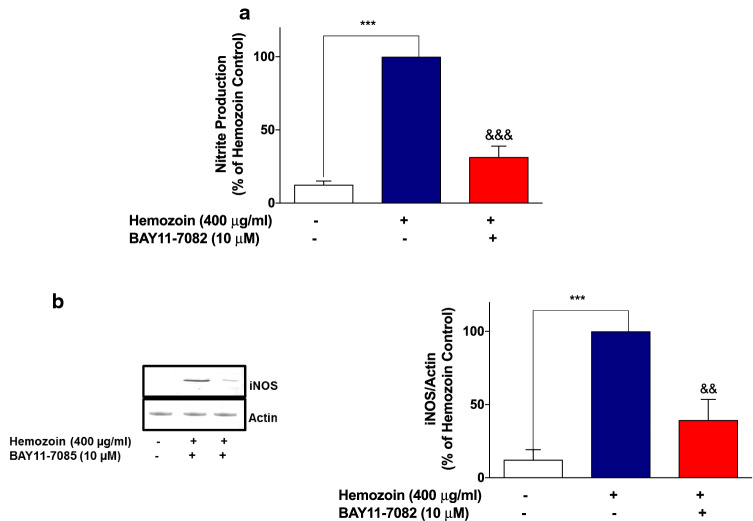
BAY11-7082 inhibited nitrite production and iNOS protein expression in sHZ-treated microglia. BV2 cells were treated with sHZ (400 µg/ml) in the presence or the absence of BAY11-7082 for 24 h. Subsequently, culture supernatants and cytoplasmic lysates were collected and analyzed for **a** nitrite production and **b** iNOS protein expression using ELISA and western blot. Actin was used as a loading control. All values are expressed as mean ± SEM for three independent experiments. Data were analyzed using one-way ANOVA with post hoc Tukey’s multiple comparison test. ***p < 0.001 compared with untreated control. ^&&^*p* < 0.01, ^&&&^*p* < 0.001, Hemozoin alone versus BAY11-7082 + Hemozoin

Having established that sHZ may be producing activation of NF-κB in BV-2 microglia, we confirmed whether the transcription factor plays any direct role in sHZ-induced release of pro-inflammatory mediators in BV-2 microglia. To evaluate this, we treated the cells with a known potent inhibitor of NF-κB, BAY11-7082 (10 µM) prior to stimulating the cells with sHZ. Interestingly, our results in Fig. 6a show that BAY11-7082 reduced sHZ-induced production of both TNFα and IL-6 in the cells, in comparison with control. Treatment with BAY11-7082 also prevented sHZ-induced production of both pro-IL-1β and IL-1β (Fig. 6b). Similar observations were made in experiments assessing the effects of BAY11-7082 on sHZ-induced increase in nitrite production and iNOS protein level (Fig. 7a, b).

**Fig. 8:**
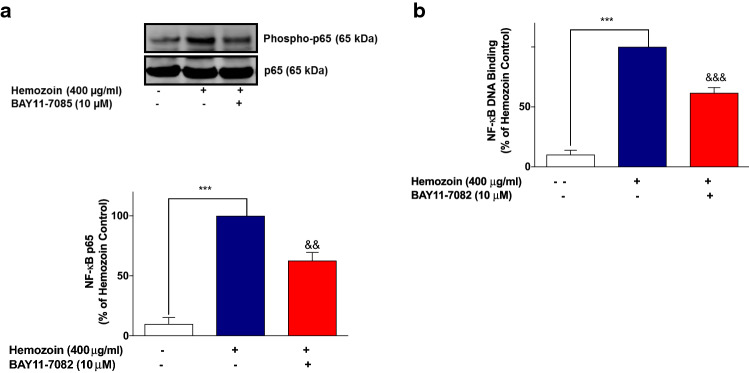
The NF-κB inhibitor BAY11-7082 prevented the phosphorylation and DNA binding of NF-κB in sHZ-stimulated BV-2 microglia. Cells were pre-treated for 30 min with BAY11-7082 (10 µM) and stimulated with 400 µg/ml of sHZ for 60 min. Subsequently, nuclear and cytoplasmic lysates were collected and subjected to ELISA-based EMSA and western blot. BAY11-7082 reduced **a** NF-κB DNA binding and **b** phosphorylation of p65 in BV2 microglia. Total p65 was used as a loading control in western blot. All values are expressed as mean ± SEM for three independent experiments. Data were analyzed using one-way ANOVA with post hoc Tukey’s multiple comparison test. ***p < 0.001 compared with untreated control. ^&&^*p* < 0.01, ^&&&^*p* < 0.001, Hemozoin alone versus BAY11-7082 + Hemozoin

These results seem to suggest that induction of neuroinflammation by sHZ is possibly mediated through activation of NF-κB. To test this hypothesis further, we showed that pre-treatment of BV-2 microglia with that of BAY11-7082 (10 µM) resulted in significant (*p* < 0.01) reduction in the phospho-p65 protein as well as DNA-bound NF-κB p65, in comparison with sHZ stimulation alone (Fig. 8a, b).

### Increases in NLRP3 Protein and Caspase-1 Activity in sHZ-Stimulated BV-2 Cells were Reduced in the Presence of BAY11-7082

**Fig. 9:**
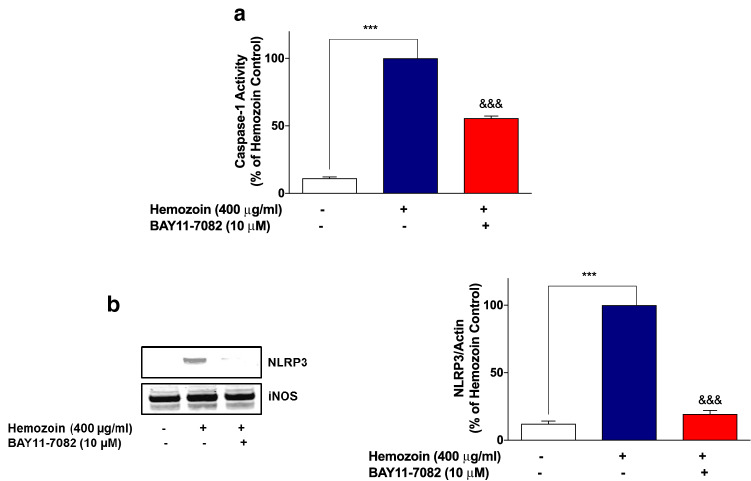
Increases in caspase-1 activity and NLRP3 protein in sHZ-stimulated BV-2 cells were reduced in the presence of BAY11-7082. BV2 cells were pre-treated with BAY11-7082 followed by the stimulation with sHZ 400 µg/ml for 24 h. Later, **a** caspase-1 activity was measured using inflammasome Glo assay and **b** cytoplasmic lysates were used to detect NLRP3 expression using western blot. Actin was used as a loading control in the western blot. All values are expressed as mean ± SEM for three independent experiments. Data were analyzed using one-way ANOVA with post hoc Tukey’s multiple comparison test. ***p < 0.001 compared with untreated control. ^&&&^*p* < 0.001, Hemozoin alone versus BAY11-7082 + Hemozoin

Based on our results showing that BAY11-7082 prevented the release of pro-IL-1β/IL-1β in sHZ-stimulated BV-2 microglia, we became interested in determining whether this inhibitor could prevent the earlier observed increase in NLRP3 protein and caspase-1 activity following the exposure of BV-2 cells to sHZ. Results in Fig. 9a show that in the presence of sHZ (400 µg/ml), there was a significant (*p* < 0.001) increase in caspase-1 activity, in comparison with untreated control. However, in the presence of BAY11-7082 (10 µM), activity of caspase-1 was significantly (*p* < 0.001) diminished, when compared with sHZ stimulation alone. Similar trends were observed in western blotting experiments to detect NLRP3 protein (Fig. 9b); pre-treatment of BV-2 microglia with BAY11-7082 (10 µM) prior to stimulation with sHZ (400 µg/ml) resulted in a significant reduction in NLRP3 protein.

### Release of Pro-IL-1β/IL-1β, Nitrite as well as iNOS Protein Expression was Attenuated by the NLRP3 Inhibitor CRID3 in sHZ-Stimulated BV-2 Microglia

**Fig. 10:**
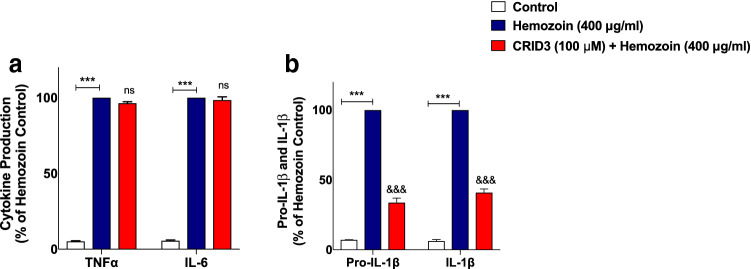
Release of pro-IL-1β/IL-1β was blocked by the NLRP3 inhibitor CRID3 in BV-2 microglia stimulated with sHZ, while TNFα and IL-6 were unaffected. BV2 cells were pre-treated with CRID3 (100 µM) prior to exposing the cells to 400 µg/ml of sHZ. Culture supernatants were collected and analyzed for **a** TNF-α, IL-6, and **b** pro-IL-1β and IL-1β using ELISA. All values are expressed as mean ± SEM for three independent experiments. Data were analyzed using one-way ANOVA followed by a post hoc Tukey’s multiple comparison test. ***p < 0.001 compared with untreated control. ^&&&^*p* < 0.001, Hemozoin alone versus CRID3 + Hemozoin. *ns* not significant

Having made the interesting observation that sHZ could be inducing neuroinflammation in BV-2 cells by activating NLRP3 inflammasome, we wanted to know the extent of its direct involvement in the release of pro-inflammatory mediators in BV-2 microglia stimulated with sHZ. Pre-treatment of BV-2 microglia with NLRP3 inhibitor CRID3 (100 µM) prior to stimulation with sHZ did not affect the levels of TNFα and IL-6 in culture supernatants, when compared with sHZ stimulation alone (Fig. 10a). Interestingly, in the presence of CRID3 (100 µM), there was a marked and significant (*p* < 0.001) reduction in the levels of pro-IL-1β and IL-1β in culture supernatants, in comparison with sHZ (400 µg/ml) alone (Fig. 10b).

**Fig. 11:**
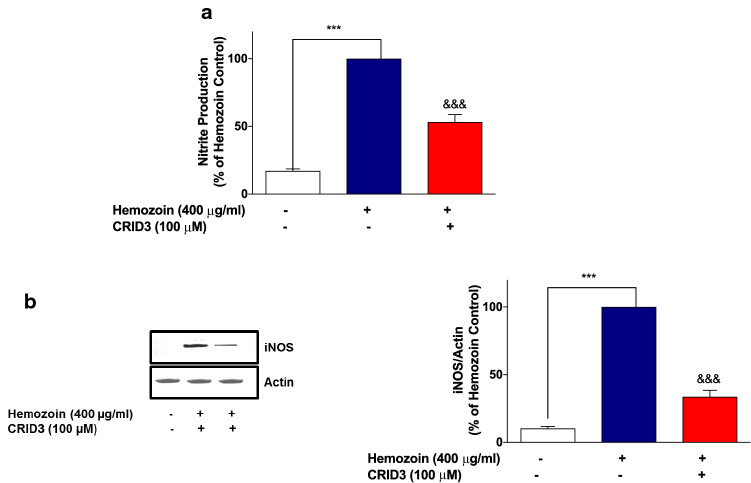
CRID3 inhibited nitrite production and iNOS protein expression in sHZ-treated microglia. BV2 Cells were treated with sHZ (400 µg/ml) in the presence or absence of CRID3 (100 µM) for 24 h. Subsequently, culture supernatants and cytoplasmic lysates were collected and analyzed for **a** nitrite production and **b** iNOS protein expression using ELISA and western blot. Actin was used as a loading control. All values are expressed as mean ± SEM for three independent experiments. Data were analyzed using one-way ANOVA with post hoc Tukey’s multiple comparison test. ***p < 0.001 compared with untreated control. ^&&&^*p* < 0.001, Hemozoin alone versus CRID3 + Hemozoin

In separate experiments to determine whether incubation of BV-2 cells with CRID3 could have any effect on sHZ-induced nitrite/iNOS levels, results show that when cells were stimulated with sHZ (400 µg/ml), there were significant increases in both nitrite production and iNOS protein level. These increases were significantly attenuated when cells were pre-treated with CRID3 (100 µM) (Fig. 11a, b).

**Fig. 12:**
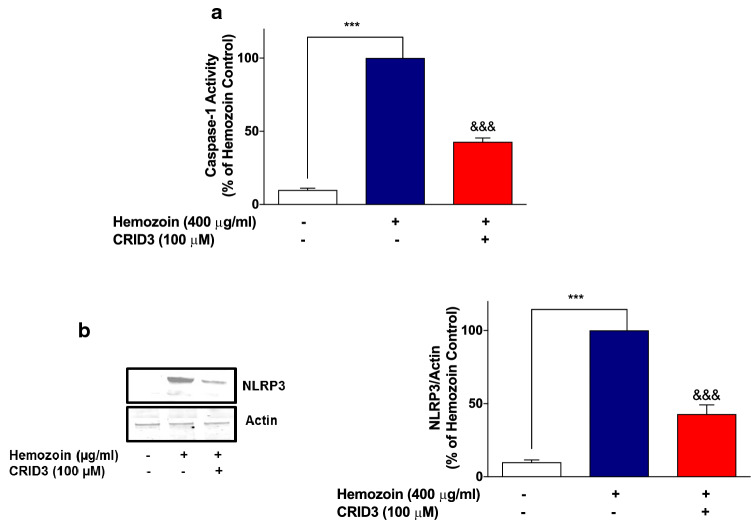
Increases in caspase-1 activity and NLRP3 protein in sHZ-stimulated BV-2 cells were reduced in the presence of NLRP3 inhibitor. BV2 cells were pre-treated with CRID3 (100 µM) followed by the stimulation with sHZ 400 µg/ml for 24 h. **a** Caspase-1 activity was measured using inflammasome Glo assay and **b** cytoplasmic lysates were used to detect NLRP3 expression using western blot. Actin was used as a loading control in the western blot. All values are expressed as mean ± SEM for three independent experiments. Data were analyzed using one-way ANOVA with post hoc Tukey’s multiple comparison test. ***p < 0.001 compared with untreated control. ^&&&^*p* < 0.001, Hemozoin alone versus CRID3 + Hemozoin

Finally, sHZ-induced increase in NLRP3 protein and caspase-1 activity was shown to be significantly (*p* < 0.001) inhibited in the presence of CRID3 (100 µM) (Fig. 12a, b), further confirming that the pigment activates NLRP3 as part of its mechanisms for inducing neuroinflammation in BV-2 microglia.

### Exposure of Differentiated 3D ReNcell VM Human Neural Cells to sHZ Promotes Reduced Viability, Increased ROS Generation, and Elevated Caspase-6 Activity

**Fig. 13:**
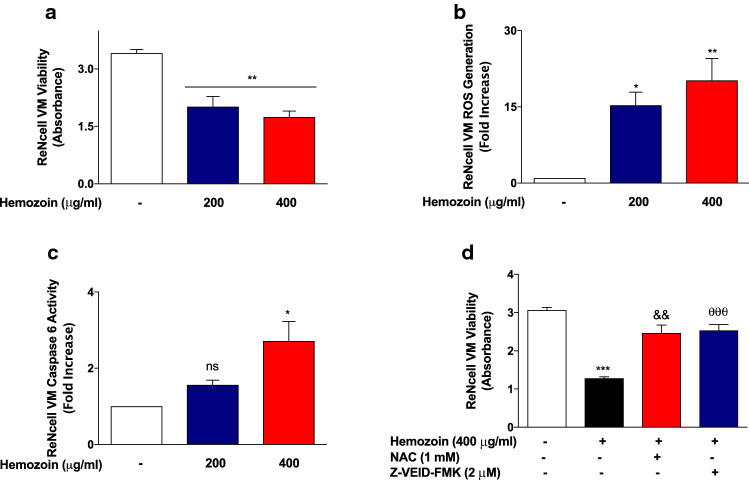
sHZ-reduced viability increased ROS and caspase-6 levels in differentiated 3D ReNcell VM human neural cells. Cells were treated with 200 and 400 µg/ml of sHZ for 24 h. **a** Cell viability was assessed using the MTT cell viability assay. **b** Generation of intracellular reactive oxygen species (ROS) levels was performed using the DCFDA-cellular reactive oxygen species detection assay kit. **c** Caspase-6 levels were measured from the cytoplasmic lysates using caspase-6 activity assay. **d** MTT assay showing that pre-treatment of differentiated 3D ReNcell VM human neural cells with NAC and Z-VEID-FMK prevented sHZ-induced neural damage. All values are expressed as mean ± SEM for three independent experiments. Data were analyzed using one-way ANOVA with post hoc Tukey’s multiple comparison test. *p < 0.05, **p < 0.01, ***p < 0.001 compared with untreated control. ^&&^*p* < 0.01, Hemozoin alone versus NAC + Hemozoin. ^θθθ^*p* < 0.001, Hemozoin alone versus Z-VEID-FMK +Hemozoin

Experiments to determine whether sHZ could produce direct neural damage revealed that treatment of differentiated 3D neural cells with 200 and 400 µg/ml of sHZ resulted in reduced cellular viability. As shown in Fig. 13a, incubation with 200 µg/ml of sHZ resulted in cellular viability of ~ 60% when compared with untreated cells, while treatment with 400 µg/ml of the pigment resulted in ~ 51% viability, in comparison with untreated cells. Interestingly, reduced neural cell viability was accompanied by significant (*p* < 0.05) elevation in the generation of cellular reactive oxygen species (Fig. 13b).

Based on results showing neurotoxic effects of sHZ, we explored the possible impact of the pigment on caspase-6, which has been linked to apoptosis-mediated neurodegeneration in AD. Results in Fig. 13c show that at 200 µg/ml, sHZ induced a modest ~ 1.6-fold increase in the activity of caspase-6 in differentiated ReNcell VM human neural cells. On increasing the concentration of sHZ to 400 µg/ml, we observed significant (*p* < 0.05) ~ 2.7-fold increase in caspase-6 activity.

We further confirmed the roles of ROS generation and caspase-6 activity in sHZ-induced neurotoxicity by determining its effect on neural viability in the presence of *N*-acetylcysteine (ROS inhibitor) and Z-VEID-FMK (casapse-6 inhibitor). Results of MTT cell viability assay in Fig. 13c show that exposure of sHZ (400 µg/ml) resulted in significant (*p* < 0.001) reduction in the viability of differentiated ReNcell VM cells. Interestingly, the degree of neurotoxicity was significantly reduced (*p* < 0.01) in cells pre-treated with *N*-acetylcysteine (1 mM) or Z-VEID-FMK (2 µM).

Analysis of culture supernatants from differentiated ReNcell VM cells following incubation with hemozoin (200 and 400 µg/ml) for 24 h did not reveal significant increase in the levels of TNFα, IL-6, IL-1β, and nitrite (Supplementary Data 2).

## Discussion

Studies have suggested that the malaria pigment, hemozoin plays a significant role in the immunological responses in the disease. By extension, hemozoin has been strongly linked to the neurological sequelae in cerebral malaria. In this study, we investigated the effects of a synthetic hemozoin on the activation of BV-2 microglia, with subsequent induction of neuroinflammation, and showed that incubating BV-2 microglia with hemozoin resulted in the release of pro-inflammatory cytokines TNFα, IL-6, IL-1β. We also provide evidence that hemozoin-induced activation of BV-2 microglia is accompanied by iNOS-mediated NO production. Earlier, Coban et al. reported that hemozoin activated microglia cells to induce the up-regulation of genes such as lipocalin 2, MIP-1α, and IL-6 (Coban et al. 2007). Furthermore, hemozoin was shown to induce inducible iNOS-mediated NO production from macrophages (Jaramillo et al. 2003). These reports, coupled with our observations, provide significant evidence demonstrating that macrophages/microglia are activated in the presence of hemozoin, resulting in the release of pro-inflammatory proteins.

Reports have suggested that the release of mature IL-1β from pro-IL-1β in neuroinflammation is mediated through NLRP3 inflammasome activation (Freeman et al. 2017; Yang et al. 2018). Based on our results showing an increase in the secretion of pro-IL-1β/IL-1β in BV-2 cells treated with hemozoin, we carried out investigations which showed that the synthetic form of the pigment increased the levels of NLRP3 protein, accompanied by an increase in caspase-1 activity in the cells. These results seem to suggest that the ability of hemozoin to induce the secretion of IL-1β in BV-2 cells can be partly explained by its ability to activate the NLRP3 inflammasome, and subsequently activating caspase-1. This outcome appears to be similar to earlier reports of activation of NLRP3 and subsequent release of IL-1β in vivo and in vitro in macrophages (Griffith et al. 2009; Dostert et al. 2009; Strangward et al. 2018). However, this is the first evidence demonstrating activation of the NLRP3 inflammasome and subsequent production of IL-1β in BV-2 microglia by hemozoin.

Some studies have shown that hemozoin produces inflammation through the activation of toll-like receptors, specifically TLR2 and TLR9 (Coban et al. 2007; Jaramillo et al. 2003; Freeman et al. 2017). Activation of signaling pathways involving TLR2 and TLR9 by various ligands has been shown to result in subsequent activation of NF-κB signaling pathway (Yang et al. 2018; Coban et al. 2005; Liu et al. 2018; Hou et al. 2017; Li et al. 2017). Our investigations revealed that synthetic hemozoin used in this study induced the phosphorylation of the p65 sub-unit followed by its DNA binding. We also showed that hemozoin-induced release of TNFα, IL-6, as well as iNOS-mediated NO production in BV-2 microglia was inhibited in the presence of BAY11-7082. Furthermore, BAY11-7082 attenuated hemozoin-induced phosphorylation of p65 and its DNA binding. We further demonstrated that BAY11-7082 attenuated hemozoin-induced increase in NLRP3 activation, caspase-1 activity, and the resulting release of IL-1β in BV-2 microglia. We propose for the first time that while the membrane receptors involved are still unknown, synthetic hemozoin induces the release of pro-inflammatory mediators such as TNFα, IL-6, iNOS, and activates NLRP3 inflammasome to release IL-1β in BV-2 microglia through mechanisms involving the activation of NF-κB signaling pathway. This is an intriguing outcome; cross-talk mechanisms between NF-κB and NLRP3 in innate immunity have been extensively reviewed by Kersse et al. (2011). These authors proposed that activation of NF-κB and its transcriptional activity results in the direct secretion of IL-1β. Activation of NF-κB also triggers NLRP3 through caspase-1, with subsequent release of mature IL-1β. Interestingly, earlier studies by Jaramillo et al. showed that hemozoin induced NF-κB-mediated iNOS/NO production in peripheral macrophages (Jaramillo et al. 2003). To our knowledge, this is the first report showing NF-κB activation as a mechanism involved in hemozoin-induced NLRP3 inflammasome activation in BV-2 microglia.

The NLRP3 inhibitor CRID3 did not affect hemozoin-induced TNFα and IL-6 production, but suppressed the release of IL-1β from BV-2 microglia. This is an interesting outcome which seems to suggest that NLRP3 activation is required for hemozoin-induced release of IL-1β, but not TNFα or IL-6. This, however, does not explain results showing that increases in both iNOS protein levels and NO production by hemozoin were inhibited by CRID3 because production of iNOS, TNFα, and IL-6 is normally under transcriptional control of NF-κB. Consequently, investigations are needed to determine whether hemozoin produces NLRP3-dependent increase in iNOS protein and NO production through other mechanisms that are coupled to NF-κB.

The ReNcell VM is a human neural progenitor cell line which readily differentiates into neurons, astrocytes, and oligodendrocytes; a process we have earlier reported (Velagapudi et al. 2018), and has been proposed as a model of neurodegenerative diseases such as AD (Choi et al. 2014). Furthermore, it is now well established that neuroinflammation plays a significant role in neurodegeneration. As a result of induction of neuroinflammation by hemozoin, we became interested in evaluating whether the pigment could induce neuroinflammation-mediated neural damage to differentiated ReNcell VM cells. Co-culture experiments revealed that exposure of BV-2 microglia to hemozoin resulted in damage to ReNcell VM cells, accompanied by elevated levels of neurotoxic mediators (NO, TNFα, and IL-1β). These observations indicate that exposure to hemozoin seem to induce neuroinflammation, with subsequent neuronal loss in cerebral malaria.

We also exposed hemozoin directly to differentiated ReNcell VM cells; a neural system composing of astrocytes which are now known to be involved in neuroinflammation and neurotoxicity (Colombo and Farina 2016; Cekanaviciute and Buckwalter 2016; Neal and Richardson 2018), as well as mature neurons. In this study, we showed for the first time that hemozoin-induced damage to differentiated human neural progenitor cells is accompanied by marked induction of oxidative stress. These results are consistent with previous reports showing that oxidative stress mediates toxicity to human neural progenitor cells (Rharass et al. 2017; Chang et al. 2013). Remarkably, our experiments showed hemozoin induced caspase-6 activation in differentiated ReNcell VM human neural progenitor cells. We confirmed the roles of oxidative stress and caspase-6 activation in hemozoin-induced neural damage by demonstrating a prevention of hemozoin-induced neural damage in the presence of N-acetylcysteine and Z-VEID-FMK, which are ROS and caspase-6 inhibitors, respectively. These new observations are quite interesting as oxidative stress has been linked to the activation of caspase-6 in neurodegeneration (Islam et al. 2019). Activation of caspase-6 has been linked to the neurobiology of neurodegeneration (Ramcharitar et al. 2013; LeBlanc 2013; Wang et al. 2015; Foveau et al. 2016). Consequently, we propose that malarial hemozoin promotes oxidative stress- and caspase-6-mediated neuronal loss, which explains the presence of neurological sequelae in cerebral malaria.

ReNcell VM neural cells are able to differentiate into astrocytes, which have been implicated in neuroinflammation. However, our experiments revealed that hemozoin did not induce the production of pro-inflammatory mediators (TNFα, IL-6, IL-1β, and NO) in these cells following differentiation. The failure of hemozoin to induce neuroinflammation in this system could be attributed to the limited role of astrocytes in neuroinflammation-mediated neuronal damage in this model. Experiments are required to further investigate the role of astrocytes generated as a result of ReNcell VM differentiation in producing neuroinflammation-mediated neurotoxicity following stimulation with hemozoin.

In conclusion, our study has demonstrated that the synthetic form of hemozoin is able to activate neuroinflammation in BV-2 microglia through mechanisms that appear to be centrally controlled through NF-κB signaling pathway, and partly through the activation of NLRP3 inflammasome activation. This pigment has also been shown to promote marked neuronal damage through the induction of neuroinflammation, oxidative stress, and activation of caspase-6. These actions of hemozoin on the microglia and differentiated neural cells have thrown more light on the possible role of the natural malarial hemozoin in the neurobiology of cerebral malaria. This study has also proposed potential molecular targets for new drugs that could serve as adjunctive treatment in cerebral malaria. The synthetic form of hemozoin was employed in this study; further studies are focusing on induction of neuroinflammation and neurodegeneration by hemozoin isolated from *P. falciparum*. In particular, the potential link between hemozoin production in cerebral malaria and long-term risk of neurodegeneration needs to be investigated.

## Electronic supplementary material

Below is the link to the electronic supplementary material.

Supplementary material 1 (PPTX 260 kb)
