# Supplementary figures and images for: Induction of Neuroinflammation and Neurotoxicity by Synthetic Hemozoin
Source: Cell Mol Neurobiol. 2019 Jul 22;39(8):1187–200. doi: 10.1007/s10571-019-00713-4 (PMC6764936; doi:10.1007/s10571-019-00713-4)

## Slide 1
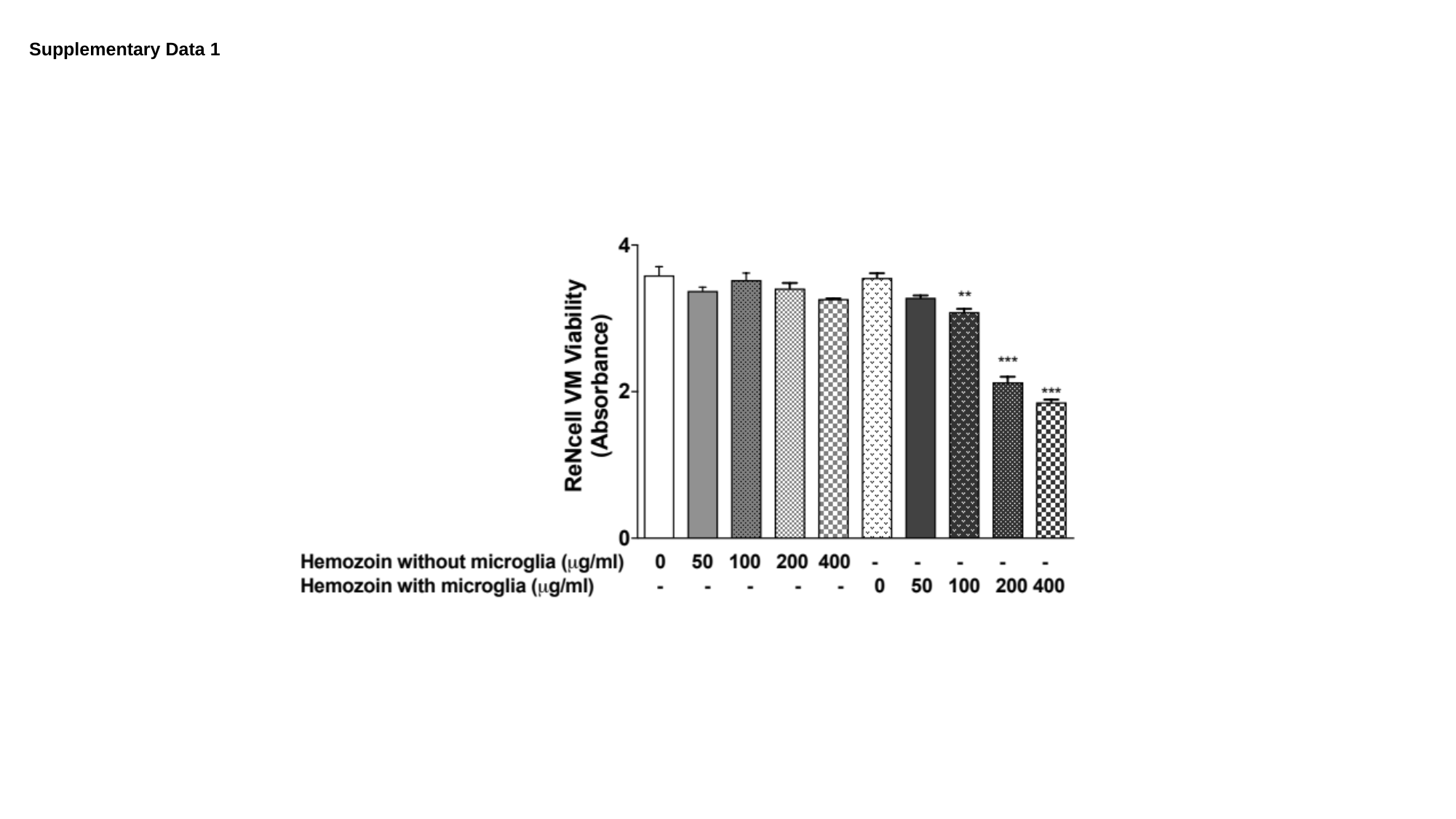

Supplementary Data 1

## Slide 2
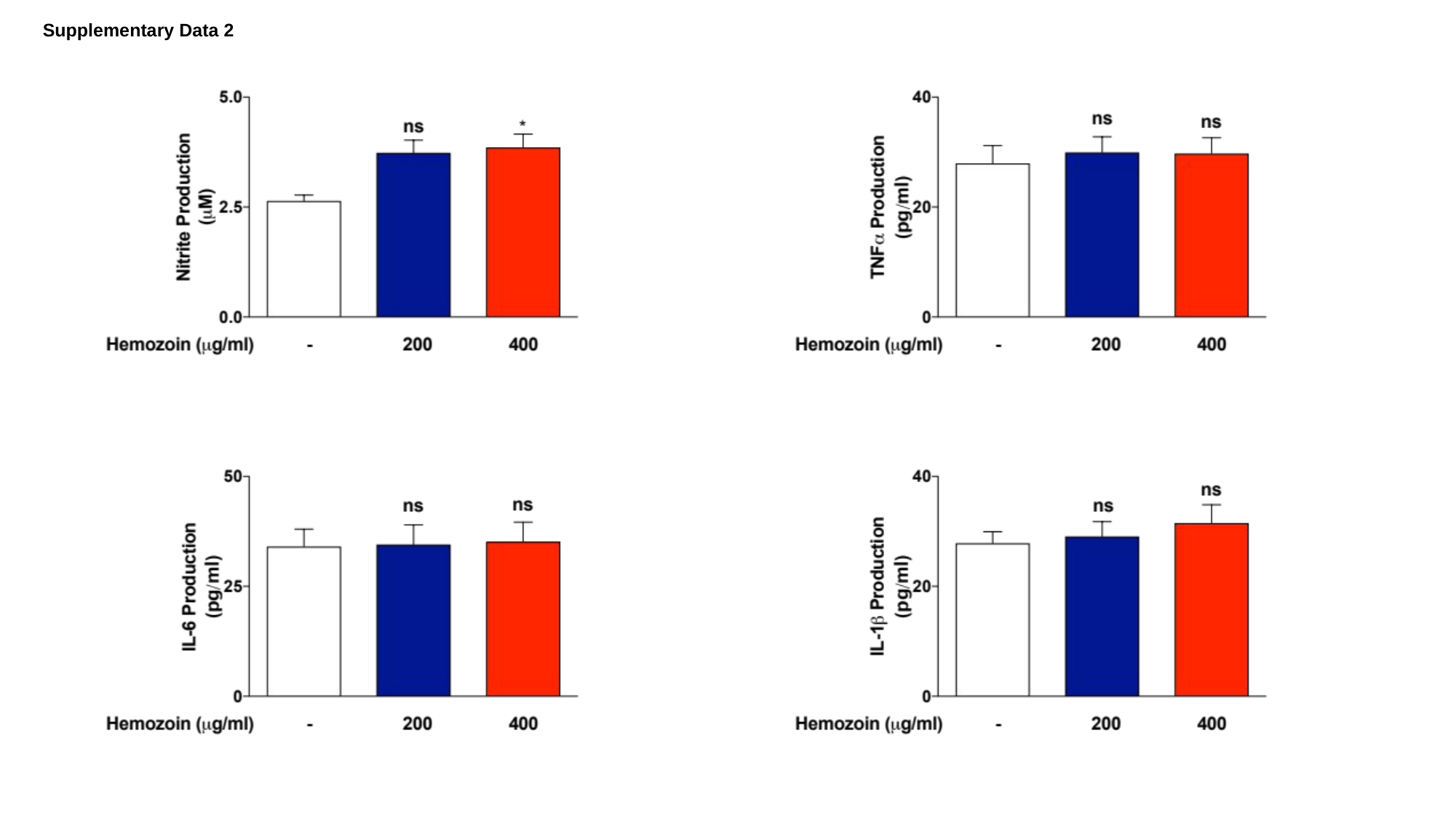

Supplementary Data 2

Supplement: Supplementary file 1 — Supplementary material 1 (PPTX 260 kb) [file 10571_2019_713_MOESM1_ESM.pptx]
